# Features of cardiac remodeling in Patients with Acute Coronary Syndrome Complicated with Rheumatoid Arthritis

**DOI:** 10.1038/s41598-017-11123-1

**Published:** 2017-08-31

**Authors:** Lili Pan, Tian Wang

**Affiliations:** 1 Department of Rheumatology and Immunology, Beijing Anzhen Hospital, Capital Medical University, Beijing, 100029 China; 2 Beijing Institute of Heart, Lung, and Blood Vessel Diseases, Beijing, 100029 China

## Abstract

Cardiovascular diseases are important factors to increased morbidity and mortality in patients with rheumatoid arthritis (RA). The aim of this study is to investigate the effects of RA on cardiac remodeling in patients with acute coronary syndrome (ACS). Sixty-one patients with ACS complicated with RA (RA group) and 55 age- and sex-matched patients with ACS without RA (control group) were enrolled. We compared the parameters of laboratory and echocardiogram across the 2 groups. Levels of serum brain natriuretic peptide in patients with RA were significantly higher than control group. Prevalence of left ventricular hypertrophy (LVH), and LV diastolic dysfunction (E/A < 1) were significantly higher in the RA patients, while the LV ejection fraction (EF%) was significantly lower in RA patients. Incidence of tricuspid regurgitation and pulmonary regurgitation were significantly higher in ACS patients with RA than in the ACS patients without RA. In RA group, levels of serum high density lipoprotein cholesterol were negatively correlated with C reactive protein (CRP), EF% was also negatively correlated with CRP. The prevalence of LVH and mitral regurgitation showed positive correlations with ESR. Early intervention for controlling the inflammation associated with RA can play a significant role in preventing cardiac remodeling in ACS patients.

## Introduction

Rheumatoid arthritis (RA) is an autoimmune disease characterized by the participation of many inflammatory cytokines, which can cause serious joint deformity and systemic organ impairment. RA is considered an independent cardiovascular risk factor^1^. Patients with RA have an excess risk of heart failure and related poor prognosis compared with the general population. Recent research findings suggest that patients with RA have risk of cardiac involvements twice or thrice that of patients without RA. The risk even increases two years before the diagnosis of RA was established^2, 3^. RA is strictly related to accelerated atherosclerotic process, and higher prevalence of cardiovascular disease (CVD) in RA patients could be explained by other mechanisms than the classic atherosclerotic risk factors^4^. Chronic inflammation plays a vital role in the high risk of CVD in RA patients, the abnormal immune responses and chronic inflammation in CVD and RA share many common similarities. European League Against Rheumatism (EULAR) has recommended RA should be regarded as a condition associated with higher risk for CVD, and adequate control of disease activity is necessary to lower the CVD risk^5^. Abundant research findings have suggested that RA causes both cardiac morphology and function changes. Some studies documented worse left ventricular systolic function and diastolic function in patients with RA^6, 7^. Acute coronary syndrome (ACS) is an urgent cardiovascular event, studies have shown a higher incidence myocardial infarction, congestive heart failure, and coronary deaths in patients with RA than in the general population^8^. However, research findings on the effects of RA on cardiac structure and function of the patients with ACS are limited. The research described here investigated cardiac remodeling in patients with ACS complicated with RA through analysis of laboratory parameters and echocardiogram.

## Materials and Methods

### Subjects

A retrospective cross-sectional study was performed. Sixty-one patients with ACS complicated with RA (RA group) were recruited from January 2012 to December 2015 in Beijing Anzhen Hospital, Capital Medical University, Beijing, China. All patients fulfilled the classification criteria of RA revised by American College of Rheumatology in 1987^9^. 55 age- and sex-matched ACS patients without RA were recruited as controls. All patients had acute myocardial infarction (AMI) and unstable angina pectoris (UAP), which were diagnosed based on symptoms, physical examination, electrocardiograms, myocardial enzyme determination, echocardiography, and coronary angiography according to the current European Society Cardiology guidelines^10, 11^.

The following medical examinations were performed for all subjects after hospitalization: height, weight, Body Mass Index (BMI) calculated as weight to square of height ratio, and blood pressure. Cardiovascular risk factors include smoking (ever and current), family history of CVD, hypertension, dyslipidemia and diabetes mellitus. Traditional cardiovascular risk factors were defined as: cigarette smoking (in the previous 10 years), hypertension (systolic blood pressure >140 mmHg or diastolic blood pressure >90 mmHg), diabetes mellitus (fasting serum glucose >126 mg/dl or use of antidiabetic medications), hyperlipidemia (total cholesterol >200 mg/dL, low–density lipoprotein (LDL-C) > 130 mg/dL, triglycerides >130 mg/dL, or high–density lipoprotein (HDL-C) <40 mg/dL).

Patients with the following conditions were excluded from the study: other autoimmune disease, liver and kidney diseases. The informed consent was obtained from all participants and/or their legal guardians. The study was conducted in accordance with the Declaration of Helsinki and this study was approved by the Ethics Committee of Beijing Anzhen Hospital (approval number: 2016012X), Capital Medical University.

## Methods

For each subject, 4 ml venous blood was drawn in the morning next after a 12-hour fast. The blood was then placed in a tube without anticoagulant, and the serum was collected from the coagulated blood and centrifuged 3000 rpm/min for 5 min. Serum triglyceride (TG), total cholesterol (TC), high density lipoprotein cholesterol (HDL-C), low density lipoprotein cholesterol (LDL-C), high sensitive C reactive protein (hs-CRP), homocysteine (HCY), and serum brain natriuretic peptide (BNP) were tested for all patients using an automatic biochemical analyzer (Hitachi 7600-120, Tokyo, Japan).

Echocardiograms were obtained on all the patients preoperatively with use of the Vivid 7 cardiac ultra-sonography system (GE Ving Med Ultrasound AS; Horten, Norway). Left ventricular end diameter (LVEDd), interventricular septal thickness (IVSd), and left ventricular posterior wall thickness (PWTd) were measured by taking the mean values of three continuous heartbeat cycles in the expiratory state. Then, left ventricular mass (LVM) (g) was calculated with formula: 1.04 × 0.8 × [(IVSd + PWTd + LVEDd)^3^−LVEDd^3^] + 0.6.

LVM index (LVMI) were calculated by adjustment for body surface area^12^. Left ventricular hypertrophy (LVH) was defined as LV mass index >95 (g/m^2^) for females and >115 (g/m^2^) for males according to echocardiography^13^. LVEF% and E/A peak values were also measured and calculated. Left ventricular diastolic function was evaluated by E/A, and is defined as E/A < 1. Furthermore, cardiac valve regurgitation was observed.

## Statistical Methods

Statistical analysis was carried out using SPSS statistical software version 16.0 (SPSS Inc, Chicago, Illinois, USA).Values are expressed as means ± standard error (means ± SEM). Differences between measured parameters in patients and controls were assessed by unpaired *t* test. The assessment of qualitative parameters was performed by χ^2^ test. A level of P < 0.05 was considered to be statistically significant.

## Results

### Comparison of biochemical parameters and cardiovascular risk factors between ACS patients with or without RA

In the RA group, 17 were male and 44 were female (age range: from 48 to 80 years; average: 66.44 ± 8.72 years). The mean duration of RA was 18.11 ± 10.74 years. 17 patients had ST-segment elevated myocardial infarction (STEMI) and 23 had non ST-segment elevated myocardial infarction (NSTEMI), 21 had UAP. In controls, 16 were male and 39 were female (age range: from 51 to 81 years; average: 67.03 ± 6.80 years). 55 patients with ACS having no RA (ACS) were recruited. Of them, 10 patients had STEMI and 18 had NSTEMI and 27 had UAP. No difference was observed in type of ACS and treatment of ACS between the 2 groups (Table 1).

**Table 1:** Comparison of general parameters between patients with ACS complicated with and without RA.

Parameter	RA (n = 61)	Control (n = 55)	*P* value
**General demographics**			
Age, years	66.44 ± 8.72	67.03 ± 6.80	0.554
Female, n (%)	44 (72.1)	39 (70.9)	0.884
**RA duration**, years	17.11 ± 10.74	—	—
**Type of ACS**			
STEMI, n (%)	17 (27.9)	10 (18.2)	0.366
NSTEMI, n (%)	23 (37.7)	18 (32.7)	0.575
UAP, n (%)	21 (34.4)	27 (49.1)	0.109
**ACS Treatment**			
Aspirin, n (%)	59 (96.7)	52 (94.5)	0.564
Statins, n (%)	55 (90.2)	47 (85.4)	0.437
β−Βlocker, n (%)	43 (70.5)	42 (76.4)	0.475
ACEI/ARB, n (%)	27 (45.9)	24 (43.6)	0.356

STEMI: ST-segment elevation myocardial infarction, NSTEMI: nonST-segment elevation myocardial infarction, UAP: unstable angina pectoris.

The biochemical parameters from the RA group and controls are shown in Table 2. Mean value of BMI in patients with RA (27.50 ± 3.53) was significantly higher than that of controls (24.84 ± 2.36) (P < 0.05). There were no significance differences in the prevalence of smoking, family history of CVD, hypertension, dyslipidemia and diabetes mellitus. No statistically significance difference in systolic or diastolic pressures was found between the two groups. No difference was observed in the levels of serum TG, TC or LDL-C; but HDL levels were significantly lower in patients with RA (0.91 ± 0.20 mmol/L) than in controls (1.10 ± 0.23 mmol/L), (P < 0.05). Serum HCY levels (17.27 ± 4.71 mmol/L) were also significantly higher in the RA group than in controls (13.16 ± 4.23 mmol/L), (P < 0.05). The BNP levels (386.31 ± 225.88 pg/ml) in RA group patients were significantly higher than those of the control group (258.43 ± 136.97 pg/ml), (P < 0.05). The serum hs-CRP levels (9.84 ± 5.50 mg/L) in the RA group was significantly higher than in the control group (4.21 ± 3.25 mg/L), (P < 0.01). The ESR level (28.35 ± 15.87 mm/1 h) in the RA group was significantly higher than in the controls (9.33 ± 3.88 mm/1 h), (P < 0.01) (Table 2).

**Table 2:** Comparison of cardiovascular risk factors and Laboratory parameters between patients with ACS complicated with and without RA.

Parameter	RA (n = 61)	Control (n = 55)	P value
**Cardiovascular risk factors**			
Smoking			
Ever, n (%)	37 (60.1)	35 (63.6)	0.398
Current, n (%)	6 (8.2)	5 (9.0)	0.774
Family History of CVD, n (%)	25 (41.0)	19 (34.5)	0.475
BMI,kg/m^2^	27.50 ± 3.53	24.84 ± 2.36	0.044
Hypertension, n (%)	35 (57.4)	31 (56.4)	0.912
Dyslipidaemia, n (%)	31 (50.8)	30 (54.5)	0.688
Diabetes mellitus, n (%)	25 (41.0)	20 (36.3)	0.61
SBP, mmHg	132.20 ± 19.68	131.37 ± 13.76	0.797
DBP, mmHg	79.73 ± 10.81	77.79 ± 8.59	0.565
**Laboratory parameters**			
TG, mmol/L	1.43 ± 0.88	1.62 ± 0.68	0.297
TC, mmol/L	4.37 ± 1.03	4.36 ± 0.84	0.964
HDL-C, mmol/L	0.91 ± 0.20	1.10 ± 0.23	0.045
LDL-C, mmol/L	2.57 ± 0.86	2.63 ± 0.67	0.689
HCY, mmol/L	17.27 ± 4.71	13.16 ± 4.23	0.022
BNP, pg/ml	386.31 ± 225.88	258.43 ± 136.97	0.043
hs-CRP, mg/L	9.84 ± 5.50	4.21 ± 3.25	0.002
ESR, mm/1 h	28.35 ± 15.87	9.33 ± 3.88	0.01

BMI: Body Mass Index, SBP: systolic blood pressure, DBP: diastolic blood pressure, TG: triglyceride, TC: total cholesterol, HDL-C: high density lipoprotein cholesterol, LDL-C: low density lipoprotein cholesterol, HCY: homocysteine, BNP: brain natriuretic peptide, hs-CRP:high sensitivity C reactive protein. ESR: erythrocyte sedimentation rate.

### ACS Patients with RA have more prevalence of cardiac morphology and function changes compared with ACS patients without RA

Table 3 shows the echocardiography characteristics of patients with ACS with and without RA complications. The prevalence of left ventricular hypertrophy (LVH) in the RA group (50.8%) was significantly higher than in the controls (29.1%), (*P* < 0.05). LVEF% in the patients in the RA group (54.86 ± 12.12%) was significantly lower than that in patients without RA (63.83 ± 5.61%), (*P* < 0.05). The proportion of patients in the RA group who had left ventricular diastolic dysfunction (E/A < 1) was significantly higher (96.7%) than that of the control group (61.6%), (*P* < 0.01). About 45.9% of patients with RA were found to have tricuspid regurgitation, which was significantly higher than the proportion in patients with ACS only (12.7%), (*P* < 0.01). About 9.8% of patients with RA were found to have pulmonary regurgitation, which was not found in the control group (*P* < 0.05). Comparison of aortic and mitral regurgitation between the two groups showed no statistically significant differences (Table 3).

**Table 3:** Comparison of echocardiography parameters between ACS patients complicated with and without RA.

Parameter	RA (n = 61)	Control (n = 55)	*P* value
LVH, n (%)	31 (50.8)	16 (29.1)	0.017
LVEF %	54.86 ± 12.12	63.83 ± 5.61	0.038
LV diastolic dysfunction, n (%)	59 (96.7)	35 (61.6)	0
Aortic Regurgitation, n (%)	13 (21.3)	14 (25.5)	0.598
Mitral Regurgitation, n (%)	24 (39.3)	19 (34.5)	0.593
Tricuspid Regurgitation, n (%)	28 (45.9)	7 (12.7)	0
Pulmonary Regurgitation, n (%)	6 (9.8)	0 (0.0)	0.017

LVH: left ventricular hypertrophy, LVEF: left ventricular ejection fraction.

### Correlations between laboratory and echocardiogram parameters with CRP or ESR in RA patients

**Figure 1:**
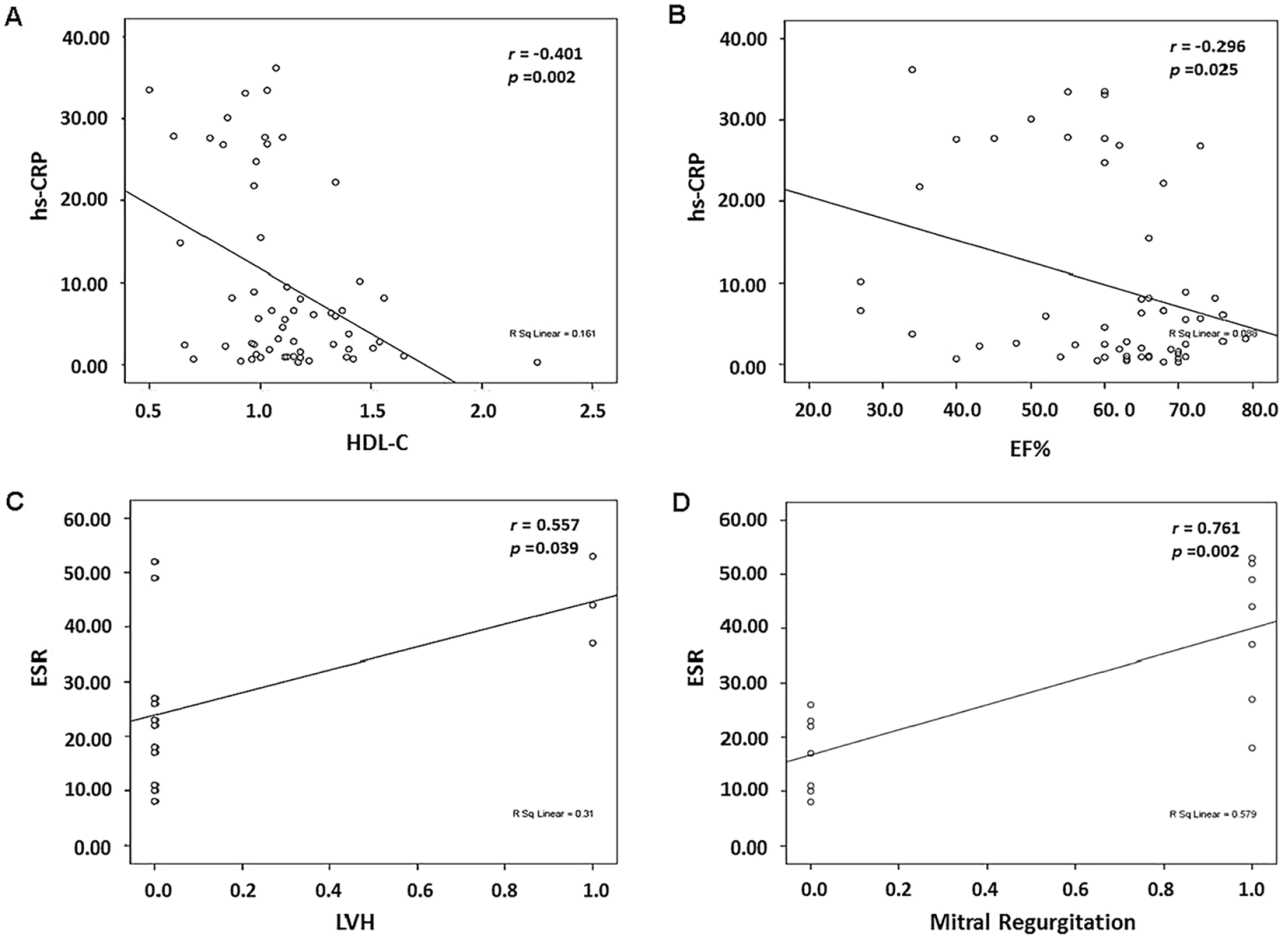
Correlations between HDL-C, EF%, prevalence of LVH and mitral regurgitation with hs-CRP or ESR. HDL-C and EF% were negatively correlated with hs-CRP. (**A**,**B**), The prevalence of LVH and mitral regurgitation shows positive correlations with ESR (**C**,**D**).

Next, we evaluated whether the laboratory and echocardiogram parameters could be correlated to CRP or ESR in the RA group. As shown in Fig. 1, the levels of serum HDL-C were negatively correlated with CRP (r = −0.401, P = 0.002), EF% were negatively correlated with CRP (r = −0.296, P = 0.025). The prevalence of LVH (r = 0.557, P = 0.039) and mitral regurgitation (r = 0.761, P = 0.002) both showed positive correlations with ESR (Fig. 1).

## Discussion

This study compared patients with acute coronary syndrome with and without RA. Traditional cardiovascular disease risk factors, laboratory index, and echocardiographic changes of cardiac structure and function of the 2 groups of patients were monitored. Patients with both RA and ACS were more likely to have left ventricular hypertrophy, tricuspid valve and pulmonary valve regurgitation, and cardiac systolic and diastolic dysfunction. In other words, ACS patients with RA were more likely to have changes in cardiac morphology and function.

In this study, among the traditional cardiovascular risk factors, markedly higher levels of BMI were found in ACS patients with RA than those without RA. Previous research has suggested that RA subjects had significantly greater BMI and fat area, and lower muscle area, muscle density, and muscle strength^14^. Another study demonstrated that an increase in RA disease activity causes an increase in BMI via an accumulation of fat tissue^15^. Previous studies have suggested that a decrease in cholesterol is the main characteristic of dyslipidemia in patients with RA^16^. This research showed no difference between the two groups with respect to the levels of serum TG, TC, or LDL-C; but HDL-C levels were significantly lower in the RA group than in controls. In RA patients, serum HDL-C levels were negatively related to RA disease activity^17^. These findings are consistent with the results of our study. Our result suggested that low HDL-C levels negatively correlated with hs-CPR in RA patients. A research showed that the high level of HCY is a predictor of atherosclerotic events in patients with RA, and closely related to cardiovascular events^18^. Our result suggests that high serum HCY level may be an important factor leading to CVD events in ACS patients with RA.

Correlations have established between RA inflammation and left ventricular remodeling^19^. Impairment of cardiac systolic and diastolic function is commonly found in patients with RA^20^. Increased left ventricular (LV) mass in patients with RA has been found to be parallel with the risks of cardiovascular morbidity and mortality^21^. Study suggested that the thickness of LV relative wall is independently associated with RA disease activity^22^. The “gold standard” for left ventricular hypertrophy (LVH) is left ventricular mass index (LVMI) on echocardiogram^23^, in our study, to exclude the influence of individual differences and accurately reflect the degree of hypertrophy, LVMI was adopted to evaluate left ventricular remodeling. Our results showed that 50.8% ACS patients with RA had LVH, which was significantly higher than those without RA (29.1%). Moreover, the prevalence of LVH exhibited a positive correlation with ESR in RA patients.

Valvular abnormalities mainly involving the mitral and aortic valve with mild to moderate regurgitation in RA patients^24^, the mechanisms might be due to the chronic inflammatory process and fibrosis of the cardiac valves^25^. Our results showed that the prevalence of mitral regurgitation was slightly more than that of the control group, but the difference was not significant between 2 groups, we found the prevalence of mitral regurgitation was correlated with ESR in RA patients. The proportion of patients with RA who had tricuspid and pulmonary valve regurgitation was significantly higher than that of the control group. This finding has not been reported in past research, and the mechanism is unclear and needs further exploration.

In RA patients, the asymptomatic reduction in cardiac systolic function is about 3 times more than non-RA population^24^. Study has suggested that RA patients with reduced LVEF% are less likely received antirheumatic drugs such as methotrexate and corticosteroids^26^. Our results showed the mean value of EF% significantly lower in patients with RA, and negatively correlated with hs-CRP. Moreover, patients with RA were more likely to present with diastolic cardiac dysfunction^27^, LV diastolic dysfunction is reported in 76% of RA patients^28^. In this study, 96.7% of the patients with RA showed decreased diastolic function, which was significantly higher than the control group (63.6%). Diastolic dysfunction in RA patients is mainly due to LV hypertrophy, interstitial fibrosis and ischaemia, but not to RA disease activity^26, 28^. The results described above indicate that RA aggravates cardiac systolic and diastolic dysfunctions in patients with ACS.

Cardiac morphology and function can be changed in patients with ACS complicated with RA. The mechanism of the cardiac function impairment, however, has not been clearly explained yet. It is currently considered that these changes may contribute to the chronic inflammatory state of RA^29^. CRP^19^, interferon (IFN) -γ^30^, and tumor necrosis factor (TNF)-α^31^ may participate in the pathological process of left ventricular remodeling in patients with RA. Administration of TNF-α antagonist can significantly be improved cardiac remodeling in patients with RA^32, 33^. It has been revealed in genetic studies that the RA-related gene HLA-DRB1 positively is related with the increasing risk of coronary events^34, 35^. It was further indicated in this present study that RA has adverse effects on cardiac morphology and function in patients with ACS.

The main limitation of this study is that it is a retrospective cross-sectional study, most of patients were hospitalized in the department of cardiology. The DAS28 score of each RA patient cannot be calculated. In this study, we evaluated the correlations between laboratory and ultrasonic parameters of cardiac morphology and function with hs-CRP or ESR in the patients with ACS complicated with RA.

In conclusion, Patients with ACS complicated with RA are more likely to be afflicted with left ventricular remodeling, cardiac systolic and diastolic dysfunctions and cardiac valve involvement. Therefore, early intervention for controlling the inflammation of RA may play a significant role in preventing and alleviating the cardiac morphological and functional changes in patients with ACS.

## References

[1] Peters MJ (2009). Does rheumatoid arthritis equal diabetes mellitus as an independent risk factor for cardiovascular disease? A prospective study. Arthritis Rheum.

[2] Bartoloni E, Alunno A, Bistoni O, Gerli R (2010). How early is the atherosclerotic risk in rheumatoid arthritis?. Autoimmun Rev.

[3] Hollan I (2013). Cardiovascular disease in autoimmune rheumatic diseases. Autoimmun Rev.

[4] Gonzalez-Gay MA, Gonzalez-Juanatey C, Martin J (2005). Rheumatoid arthritis: a disease associated with accelerated atherogenesis. Semin Arthritis Rheum.

[5] Peters MJ (2010). EULAR evidence-based recommendations for cardiovascular risk management in patients with rheumatoid arthritis and other forms of inflammatory arthritis. Ann Rheum Dis.

[6] Tomas L (2013). Left ventricular morphology and function in patients with rheumatoid arthritis. Wien Klin Wochenschr.

[7] Giles JT (2010). Left ventricular structure and function in patients with rheumatoid arthritis, as assessed by cardiac magnetic resonance imaging. Arthritis Rheum.

[8] Maradit-Kremers H (2005). Increased unrecognized coronary heart disease and sudden deaths in rheumatoid arthritis: a population-based cohort study. Arthritis Rheum.

[9] Arnett FC (1988). The American Rheumatism Association 1987 revised criteria for the classification of rheumatoid arthritis. Arthritis Rheum.

[10] Steg PG (2012). ESC Guidelines for the management of acute myocardial infarction in patients presenting with ST-segment elevation. Eur Heart J.

[11] Sianos G (2005). The SYNTAX Score: an angiographic tool grading the complexity of coronary artery disease. EuroIntervention.

[12] Totaro S, Khoury PR, Kimball TR, Dolan LM, Urbina EM (2015). Arterial stiffness is increased in young normotensive subjects with high central blood pressure. J Am Soc Hypertens.

[13] Lang RM (2005). Recommendations for chamber quantification: a report from the American Society of Echocardiography’s Guidelines and Standards Committee and the Chamber Quantification Writing Group, developed in conjunction with the European Association of Echocardiography, a branch of the European Society of Cardiology. J Am Soc Echocardiogr.

[14] Baker JF (2014). Deficits in muscle mass, muscle density, and modified associations with fat in rheumatoid arthritis. Arthritis Care Res (Hoboken).

[15] Fukuda W (2010). Contribution of rheumatoid arthritis disease activity and disability to rheumatoid cachexia. Mod Rheumatol.

[16] Myasoedova E (2010). Total cholesterol and LDL levels decrease before rheumatoid arthritis. Ann Rheum Dis.

[17] Bublikov D, Andrienko A, Nemcev A, Goryacheva K (2014). P732Interrelations of cholesterol high density lypoproteids with viscosity of blood and inflammatory activity on DAS-28-CRP scale at patients with rheumatoid arthritis. Cardiovasc Res.

[18] Jung JM (2013). Increased carotid intima-media thickness and plasma homocysteine levels predict cardiovascular and all-cause death: a population-based cohort study. Eur Neurol.

[19] Myasoedova E (2013). Brief report: rheumatoid arthritis is associated with left ventricular concentric remodeling: results of a population-based cross-sectional study. Arthritis Rheum.

[20] Garza-Garcia C (2013). Risk factors for asymptomatic ventricular dysfunction in rheumatoid arthritis patients. ISRN Cardiol.

[21] Corrao S (2015). Rheumatoid arthritis affects left ventricular mass: Systematic review and meta-analysis. Eur J Intern Med.

[22] Midtbo H (2015). Disease activity and left ventricular structure in patients with rheumatoid arthritis. Rheumatology (Oxford).

[23] Ravi S, Rukshin V, Lancaster G, Zarich S, McPherson C (2013). Diagnosis of left ventricular hypertrophy in the presence of left anterior fascicular block: a reexamination of the 2009 AHA/ACCF/HRS guidelines. Ann Noninvasive Electrocardiol.

[24] Al-Mohaissen MA, Chan KL (2016). Echocardiography in the Assessment of Patients with Rheumatologic Diseases. Curr Cardiol Rep.

[25] Corrao S (2013). Heart involvement in rheumatoid arthritis: systematic review and meta-analysis. Int J Cardiol.

[26] Vizzardi E (2012). Echocardiographic evaluation of asymptomatic patients affected by rheumatoid arthritis. J Investig Med.

[27] Aslam F, Bandeali SJ, Khan NA, Alam M (2013). Diastolic dysfunction in rheumatoid arthritis: a meta-analysis and systematic review. Arthritis Care Res (Hoboken).

[28] Vizzardi E (2016). Prognostic value of diastolic dysfunction in asymptomatic rheumatoid arthritis patients without cardiovascular risk factors. Clin Exp Rheumatol.

[29] Kojima M (2009). Psychosocial factors, disease status, and quality of life in patients with rheumatoid arthritis. J Psychosom Res.

[30] Crowson, C.S. *et al*. Rheumatoid arthritis and cardiovascular disease. *Am Heart J* **166**, 622–28 e621 (2013). 10.1016/j.ahj.2013.07.010 PMC3890244 24093840

[31] Kimura, K., Takayanagi, R., Yokoyama, H. & Yamada, Y. Theory-based analysis of anti-inflammatory effect of TNF inhibitors on rheumatoid arthritis. *Drug Metab Pharmacokinet* (2014). 10.2133/dmpk.dmpk-13-rg-090 24418824

[32] Daien CI (2013). Etanercept normalises left ventricular mass in patients with rheumatoid arthritis. Ann Rheum Dis.

[33] Kotyla PJ (2012). Infliximab treatment increases left ventricular ejection fraction in patients with rheumatoid arthritis: assessment of heart function by echocardiography, endothelin 1, interleukin 6, and NT-pro brain natriuretic peptide. The Journal of rheumatology.

[34] Paakkanen R (2012). Proinflammatory HLA-DRB1*01-haplotype predisposes to ST-elevation myocardial infarction. Atherosclerosis.

[35] Sun W, Cui Y, Zhen L, Huang L (2011). Association between HLA-DRB1, HLA-DRQB1 alleles, and CD4(+)CD28(null) T cells in a Chinese population with coronary heart disease. Mol Biol Rep.

